# Revitalizing CPAP adherence: lessons from THN study in patients with hypoglossal nerve stimulators

**DOI:** 10.3389/frsle.2024.1380373

**Published:** 2024-07-05

**Authors:** Gimbada Benny Mwenge, Jamila Bousata, Daniel Rodenstein

**Affiliations:** ^1^Center for Sleep Medicine, Cliniques Universitaires Saint Luc, Université Catholique de Louvain, Brussels, Belgium; ^2^Cliniques Universitaires Saint Luc, Université Catholique de Louvain, Brussels, Belgium

**Keywords:** CPAP, sleep apnea, adherence, target hypoglossal neurostimulation, patient-centered approach

## Abstract

**Objective:**

This retrospective study aimed to address acceptance and long-term adherence to Continuous Positive Airway Pressure (CPAP) treatment among non-responder patients to ImThera THN system who initially declined this therapy.

**Material and methods:**

We employed a structured outpatient approach to communicate THN study results, categorize initial CPAP nonadherence reasons, and encourage CPAP trials through tailored appointments. Recorded follow-ups addressed individual concerns, providing medical guidance and acknowledging person-specific challenges. Adherence data were collected using CPAP hour meters at predetermined intervals, following Belgium's social security stipulations.

**Results:**

Between July 2014 and October 2016, eleven participants, including one woman, with prior CPAP experience (average 2 months) were enrolled. Initial non-adherence was linked to ENT or psychological factors. Ten patients agreed to CPAP trials, where interventions included changing CPAP brand, pressure adjustments, mask changes, and additional measures like cognitive-behavioral therapy and nasal spray. After 1 year, mean adherence was 6.3 ± 2 h/day, and average CPAP usage duration was 8.67 ± 2.13 years. As of November 2023, eight out of eleven patients were still actively using CPAP

**Conclusion:**

In this investigation, we challenged the concept of CPAP non-adherence, highlighting evolving adherence and the significance of continuous monitoring and personalized interventions. Our findings underscore ongoing patient education, multidisciplinary support, and dynamic intervention adaptation for enhanced adherence in challenging patient populations. The results provide insights applicable to non-adherent patients with obstructive sleep apnea, emphasizing the importance of individualized care and sustained engagement for improved CPAP acceptance.

## 1 Introduction

Obstructive Sleep Apnea Syndrome (OSAS) imposes a significant global health burden, characterized by recurrent episodes of upper airway collapse during sleep, leading to intermittent hypoxemia, fragmented sleep, and excessive daytime sleepiness (Peppard et al., 2013). Continuous Positive Airway Pressure (CPAP) therapy is the gold standard treatment for OSAS, effectively mitigating symptoms and improving overall quality of life, but its efficacy heavily relies on patient adherence (Patil et al., 2024). Unfortunately, a substantial proportion of individuals struggle with long-term adherence (Barbé et al., 2001). Side effects and discomfort associated with the mask and air pressure lead many patients to discontinue CPAP (Rotty et al., 2021). Additionally, continuous education and support are crucial, as personalized approaches such as selecting appropriate masks and managing leaks can significantly improve adherence (Leemans et al., 2018). Socioeconomic barriers, physical discomfort, and patient mistrust also contribute to low adherence rates, highlighting the need for personalized medicine and patient-centered approaches (Leemans et al., 2018; Gentina et al., 2023). Multidisciplinary clinics that offer personalized treatment solutions have been shown to significantly decrease non-adherence rates (Masoud et al., 2020; Taweesedt et al., 2022). Leveraging technology, such as wearable devices and interactive education, can enhance patient adherence and outcomes in sleep medicine (Falcone et al., 2014). Moreover, close patient interactions and engagement, including regular face-to-face meetings and thorough monitoring, are critical for improving adherence and achieving long-term success in OSA treatment (Bouloukaki et al., 2014).

In clinical practice and research literature, there is often a notable emphasis on reporting positive treatment outcomes, which can obscure our understanding of the true clinical landscape and hinder efforts to develop tailored management strategies for patients who do not respond favorably to therapy (Ioannidis, 2005). While primary treatment modalities such as CPAP are extensively studied, comparatively little attention is devoted to alternative interventions and their outcomes in non-responsive patients. Among these alternative interventions, targeted hypoglossal nerve stimulation (THN) has emerged as a promising therapeutic option for individuals intolerant of or non-compliant with CPAP therapy (Mwenge et al., 2013). THN operates by delivering electrical stimulation to the hypoglossal nerve, promoting tongue protrusion, and upper airway patency during sleep. Initial studies have shown promising results in reducing the apnea-hypopnea index (AHI) and improving sleep parameters, yet the efficacy of THN remains variable across patient populations, with certain individuals failing to achieve clinically significant improvement (Schwartz et al., 2023).

It is crucial to understand why CPAP, a proven effective treatment, did not work in patients who did not respond to hypoglossal nerve stimulation (THN), in order to address clinical challenges. Although advances in CPAP technology are numerous, they have not demonstrated an overall improvement in adherence (Kim et al., 2009; Hwang et al., 2018; Benjafield et al., 2019). This retrospective study explores the dynamics of acceptance and adherence in OSA treatment, focusing on patients initially resistant to CPAP therapy who later participated in THN studies. We hypothesize that if these technological advancements are applied in a personalized manner, they could enhance adherence to CPAP therapy among initially non-compliant patients. By retrospectively examining these factors, we aim to provide valuable insights into improving treatment adherence and outcomes for patients with OSAS, ultimately contributing to enhanced clinical practices and patient care.

## 2 Material and methods

### 2.1 Study design

**Figure 1:**
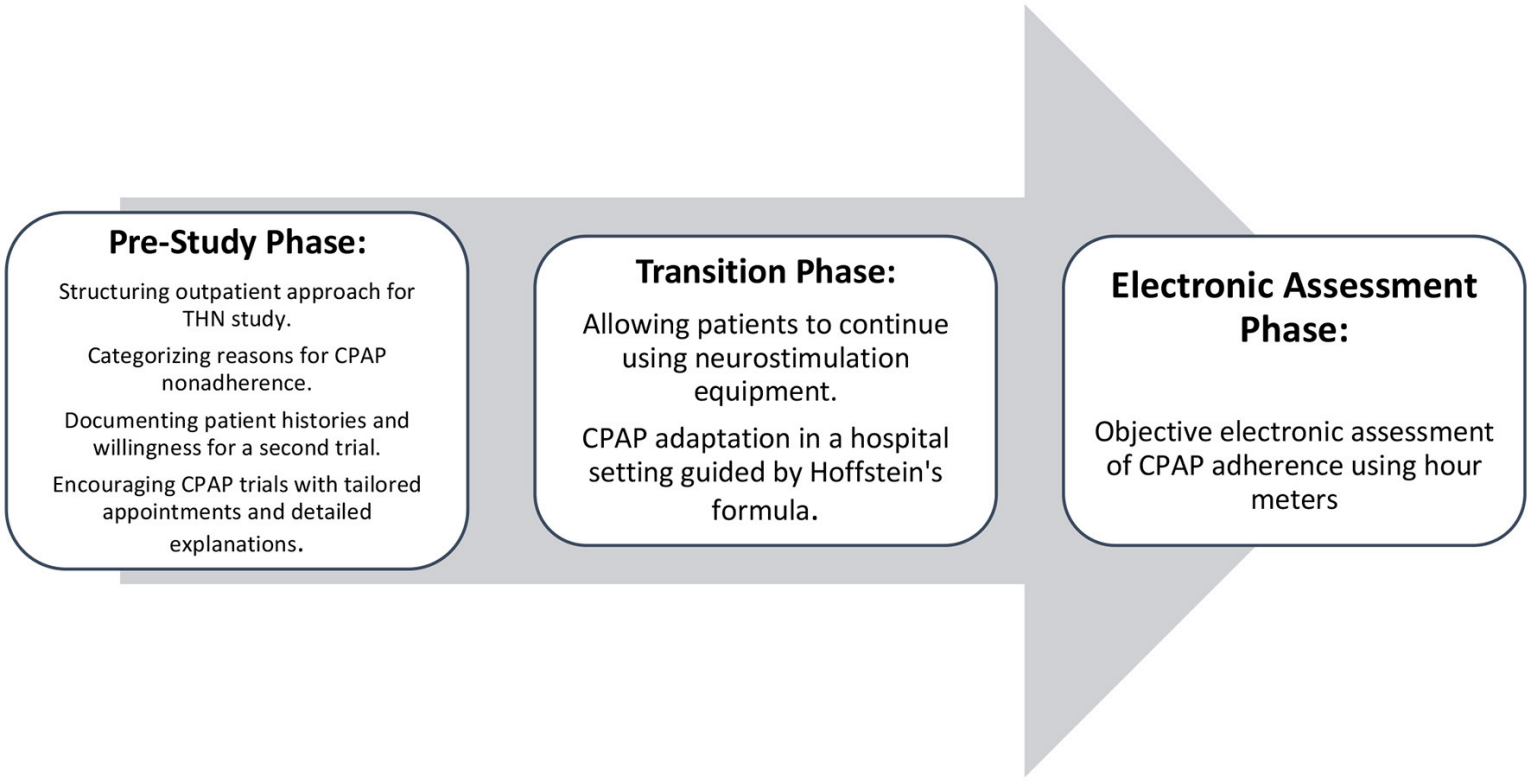
Timeline of CPAP adherence study.

This retrospective observational study assessed patients with an ImThera THN system who failed to achieve a significant reduction in the Apnea-Hypopnea Index (AHI) below 30 after 12 months. The timeline of the study is illustrated in Figure 1.

### 2.2 Patient selection

At Cliniques Universitaires Saint Luc, we conducted two studies on Targeted Hypoglossal Nerve Stimulation (THN) using the aura6000™ system, which stimulates nerve segments to activate dilator muscles. Inclusion criteria were a baseline Apnea-Hypopnea Index (AHI) of ≥20 events·h−1, refusal of CPAP therapy, BMI between 25 and 40 kg·m−2, age between 25 and 70 years, a modified Mallampati score of I to III, and palatine tonsils graded as 0, 1, or 2. Selection was independent of apnea or hypopnea indices.

**Figure 2:**
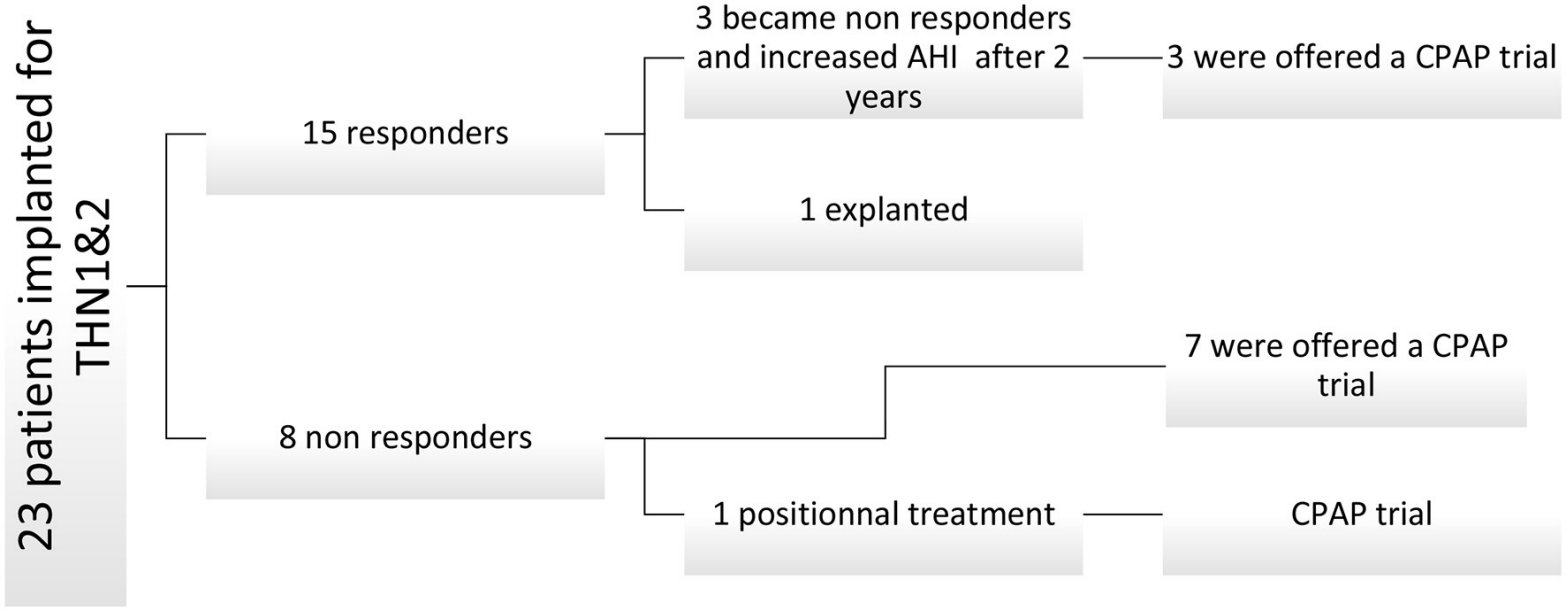
Patient inclusion.

In our center, 23 patients received THN implants. Fifteen met responder criteria, achieving an AHI below thirty events per hour. Post-THN studies revealed subjective symptoms in three patients, resulting in unsuccessful AHI reduction attempts. In three patients, stimulation ceased after 3 years due to a fibrinoleukocyte membrane hindering electrode-nerve contact. Subsequently, eleven patients explored CPAP as an alternative (see Figure 2).

### 2.3 Data collection

Data were collected from medical records, focusing on reasons for initial CPAP non-adherence and demographic characteristics. Key variables included polysomnography parameters and types of interventions for modifying CPAP and adherence.

### 2.4 Patient monitoring

During each visit and in the absence of alternatives, patients were encouraged to continue CPAP therapy. The number of outpatient visits post-study before accepting CPAP was closely monitored.

### 2.5 CPAP adaptation

Patients were hospitalized for 3 days for adaptation. They were supervised and could report any tolerance difficulties if present. Mask selection was tailored, and the effective pressure was determined by Hoffstein's formula (Lacedonia et al., 2012), starting 4 cm of water lower, with a ramp of at least 15 min. Expiratory pressure relief (EPR) was applied if necessary, either during the ramp or throughout the night. Polysomnography was utilized to assess treatment effectiveness.

### 2.6 Follow-up

Device usage data were collected at each follow-up appointment and reported after 1 year. This includes the device reading date, total usage hours, and patient compliance, calculated as total CPAP usage hours divided by days since treatment began. We also recorded the number of patients continuing treatment in December 2023 and the average compliance over 365 days.

### 2.7 Statistical analysis

The data were analyzed using descriptive statistics, including mean and median ± SD presentation. Paired *t*-tests were employed to assess baseline and follow-up differences, while Chi-squared tests were utilized for discontinuous variables. Statistical analysis was conducted using SPSS Statistics 23.

## 3 Results

Table 1 describes the characteristics of patients, baseline polysomnography data, and adherence data.

**Table 1:** Patient characteristics, baseline and THN data, and CPAP adherence.

**Baseline data**								**THN data**			**Adherence data**							
**N**	**Age Y/O**	**Sex**	**BMI**	**AH**	**ODI**	**AHIs/ AHI ns**	**AHI REM/ AHI non REM**	**AHI THN**	**AHIs/ AHI ns**	**AHI REM/ AHI non REM**	**Start of treatment**	**Reading date**	**HOURS**	**Days**	**Mean compliance**	**Adherence in 2023**	**Ongoing CPAP in 2023**	**Years of use CPAP**
1	44	M	30,1	71,7	84,2	13/60	38/76	72	15/60	33/76	1/04/16	15/12/16	385	258	1.79	4,4(in 2017)	Stop in 2017	0,75
2	53	M	25,8	70,6	75	24/48	40/75	73	2,2/72	65/75	1/02/15	31/05/16	1,246.25	450	5.5	7,2	Yes	9,76
3	54	W	39,1	79,6	75,9	8,3/60	85/78	51,4	13/28	54/50	1/03/15	13/06/16	2,820	470	6	8,1	Yes	9,68
4	68^**^	M	33,2	46,7	24,6	12/35	4/60	56	4/43	30/60	1/10/14	12/05/16	1,653	570	2.9	5,16	Yes	
5	47	M	33,5	66,6	59	**63/4**	56/67	56,8	15/43	50/58	1/03/15	24/04/16	2,142	420	5.1	6	Yes	9,69
6	43	M	26,9	61,5	44,1	**61/0,5**	60,5/61,7	26;45^*^	**20/8; 28/3**	15/25; 40/45	1/12/15	18/11/16	1,795.6	353	5.3	10	Yes	8,94
7	57	M	27,3	54	17,3	**39/2**	48/56	18;51^*^	18/1;**54/1**	10/19;4/62	1/03/15	24/10/16	3,618	603	6	6,5	Yes	9,7
8	52	M	32,4	32		29/24	18/51	40	7/31	39/40	1/01/15	18/03/16	13,786.6	442	3.1	4,8	Yes	9,86
9	55	M	29,7	30,5	28,7	**72/19**	10/20	36,4	**68/26**	9/27	1/04/15				NA	NA	No	/
10	56	M	27,4	45,1	41,6	**36/8**	9/52	45,6	**29/15**	18/56	1/07/14	6/06/16	2,700	450	NA	6	Yes	10,37
11	62	M	29,4	34,5	43,9	**33/1**	34/40	53	**43/10**	64/51	31/10/16	18/02/18	1,440	480	3	4,1(IN 2017)	Stop in 1/2021	5,1

^**^Deceased in 2018. N, patient number; Age, age of the patient; Sex, gender; Status NR refers to Non-Responder to THN therapy. W denotes patients who initially responded well but then experienced worsening con; BMI, Body Mass Index; AHI, Apnea-Hypopnea Index; ODI, Oxygen Desaturation Index; AHIs/AHI ns, AHI during supine divided by non-supine positions; AHI REM/AHI non REM, AHI during REM and non-REM sleep; AHI THN, AHI during Targeted Hypoglossal Neurostimulation; Years Of Use CPAP, total duration of CPAP usage; Start of Treatment, date when CPAP treatment commenced; Reading Date, date of the CPAP device reading; HOURS, total hours of CPAP usage recorded; Days, number of days the CPAP device was used; Compliance, adherence to CPAP treatment, calculated as hours divided by days; Adherence in 2023: Mean compliance during the last year of CPAP usage, calculated over 365 days. Ongoing CPAP in 2023, whether the patient continued CPAP treatment in 2023. The bold values represent the most significant results of the study, indicating where the data showed the highest statistical relevance. The symbol ^*^ indicates statistical significance at the 0.01 level.

In this study, eleven patients (one woman) were monitored. The baseline mean AHI was 54.9 ± 14. Six patients had positional sleep apnea, which persisted after neurostimulation. No patients had predominance associated with REM sleep events. The mean THN AHI was 47 ± 12.

All patients had ENT evaluations, including endoscopy and rhinomanometry, before the study. After detailed explanations, 10 out of 11 patients agreed to CPAP trials between July 2014 and October 2016, with a mean of 2.3 outpatient visits before acceptance. After 1 year, 8 out of 10 patients used CPAP for over 4 h. One patient stopped after 9 months, and another after 5 years, with average usage close to 4 h. As of November 2023, 8 patients continued CPAP with a mean usage of 8.67 ± 2.13 years and a mean adherence of 6.9 ± 1.4 h per day.

Table 2 patient experience and interventions in CPAP therapy: initial and second trials.

**Table 2:** Patient experience and interventions in CPAP therapy: initial and second trials.

	**Initial CPAP TRIAL**								**Second CPAP trial**					
**Sex**	**Brand**	**P (cmH2O)**	**EPR**	**Ramp**	**Mask type**	**Duration with CPAP**	**Complaints**	**Previous alternative**	**Brand**	**P (cmH2O)**	**EPR**	**Ramp**	**Mask**	**Change**
M	GK420	9	No	yes	Nasal	2 hours	ENT (nasal congestion)	UPPP	Resmed S9	7.6	1	yes	Mirage FX	Pressure−1.4 cm, + EPR
M	GK420	11	No	yes	Nasal	1 day	ENT: feeling of suffocation and patient refused the idea of CPAP	/	Resmed AutoCPAP	7 to 11 CM	No	Yes	Nasal	Switched to Auto CPAP
W	Resmed S8	10	No	Yes	Facial FP407	3 months	ENT (dryness)	/	Resmed S8	8.4	1	Yes	Nasal	Humidifier + pressure reduction + EPR
M	GK420	9	No	yes	Nasal Mirage FX	2 months	Psychological: difficulty falling back asleep with CPAP	MFT	Resmed S9	9.5	No	Yes	Nano FX mask	Change mask and machine
M	S8	10	No	yes	Nasal: Swift FX	2 months	ENT: pressure and nasal congestion	ENT surgery	Resmed S9	10	No	Yes	Nasal	Pillows mask to nasal
M	GK420	7	No	yes	Facial FP407		ENT: nasal obstruction Rhinoseptoplasty is suggested, but after surgery, the patient no longer wishes CPAP	/	Resmed S9	7	1	Yes s	Light nasal Mirage FX Nano	EPR level + mask nasal to light nasal
M	Resmed S8	8	No		Nasal: Swift FX	2 weeks	Nasal congestion, blocked nose	/	Resmed S8	8.6	No	No	Facial Quatro FX	Change to facial mask
M	GK420	7	No		Facial mask	4 years	ENT: mask-related congestion	/	GK420	9	No	Yes	Mirage FX	Change to nasal mask
M	Resmed S8	9	no		Nasal: Swift FX	3 weeks before THN	Psychological: refusal of the idea	/	Resmed S8	8 stopped	No	Yes	NA	
M	GK420	7	No		Nasal	2 weeks	ENT + insomnia ^**^, unable to tolerate, mood disturbances		Resmed S8	8.4	yes	yes	Nasal: Pillows	CBT + Hepa filter + EPR+ pillows
M	GK	12	no		Facial	1 week	Nasal congestion, blocked nose	PT	GK420	9	No	yes	Nasal: Mirage FX	Pressure reduction + EPR+ nasal spray + change mask

Gender of the participant; Brand, Brand of the CPAP machine used; P (cmH2O), Pressure setting in centimeters of water (cmH2O); EPR, Expiratory Pressure Relief (Yes/No); Ramp, Ramp feature (Yes/No); Mask Type, Type of mask used; Duration with CPAP, Duration of CPAP usage before discontinuation; Complaints, Reasons for discontinuation or complaints during CPAP usage; Previous alternative, Previous alternative or intervention tried before CPAP; Change, Changes made to the CPAP settings or mask compared to the initial trial.

Participants had prior CPAP experience averaging 2 months (range: 2 h to 4 years). They faced side effects such as nasal congestion, suffocation feelings, dryness, difficulty sleeping, pressure, and nasal obstruction. Common complaints included pressure and nasal congestion, sometimes leading to rhinoseptoplasty, and persistent nasal obstruction despite CPAP use. Psychological factors like CPAP refusal, mood disturbances, and insomnia were also observed.

During the second CPAP trial, interventions included changing CPAP brand, pressure adjustments, mask changes, cognitive-behavioral therapy, and nasal spray use. There were seven mask changes, pressure adjustments for four patients, and Expiratory Pressure Relief added for four. Most patients changed machines, with one retaining a humidifier and another switching to Autocpap.

Despite no ENT surgery, side effects either dissipated or were accepted after 2 months. Nasal masks were predominantly used, with nasal pillow masks for two patients.

## 4 Discussion

Our study aimed to explore the dynamics of acceptance and adherence in the treatment of obstructive sleep apnea (OSA), particularly focusing on patients who initially resisted CPAP therapy and subsequently participated in studies on Hypoglossal Nerve Stimulation. The hypothesis stated in the introduction was that patient-centric approaches and technological advancements could improve adherence to CPAP therapy among initially non-compliant patients.

The results demonstrated that intensive, personalized interventions could significantly enhance adherence to CPAP therapy. At the beginning of the study, the mean Apnea-Hypopnea Index (AHI) was 54.9 ± 14 among the participants, with a mean (THN) AHI of 47 ± 12 after neurostimulation, indicating a high baseline severity of OSA. Notably, six patients presented with positional sleep apnea syndrome, but positional predominance persisted post-neurostimulation. The findings that 10 out of 11 patients agreed to CPAP trials following detailed explanations highlight the importance of patient education and engagement in improving treatment acceptance.

Out of these 10 patients, 8 exhibited compliance exceeding 4 h per night after 1 year of CPAP use, showcasing a marked improvement in adherence compared to their initial resistance. This outcome suggests that non-surgical interventions, coupled with personalized support, can effectively address initial barriers to CPAP adherence in most OSA. In November 2023, eight patients continued with CPAP therapy, with a mean adherence of 6.9 ± 1.4 h per day over 8.67 ± 2.13 years, indicating sustained long-term compliance.

These findings are reinforced by the study conducted by Pépin et al. (2023), which found that 26% of individuals who initially terminated CPAP therapy resumed it within 12 months, with 65% continuing use for at least 1 year. This study also showed a significant reduction in all-cause mortality among patients who resumed CPAP therapy, underscoring the critical importance of offering a second trial of CPAP to ensure effective therapy is not withheld from those who might benefit.

However, our study observed higher adherence rates, partly due to the failure of THN treatment. The lack of success with neurostimulation likely motivated patients to reconsider and commit more seriously to CPAP therapy, resulting in improved adherence rates. This suggests that experiencing failure with an alternative treatment can drive patients to engage more diligently with CPAP therapy, as evidenced by the improved adherence in our study. Most of these patients had already tried other alternatives without success, and the severity of their sleep apnea syndrome offered no other viable options. Their commitment to neurostimulation devices, despite the challenges, underscored the complexity of their conditions and the critical need for effective solutions to their sleep apnea struggles. This may also explain the consistent daily use once the treatment was accepted (El Dib et al., 2015).

The detailed exploration of reasons for initial CPAP discontinuation, such as side effects and discomfort, is consistent with previous findings in the literature (Rotty et al., 2021). Our interventions, which included changing CPAP brands, adjusting pressures, switching masks, and implementing additional measures like cognitive-behavioral therapy and nasal sprays, were effective in mitigating these issues (Ryan et al., 2009; Genta et al., 2020). This aligns with the broader literature emphasizing the importance of personalized approaches and continuous patient support to improve CPAP adherence (Taweesedt et al., 2022).

Unexpectedly, despite no ENT surgery being performed, side effects dissipated or were accepted after 2 months of CPAP use during the second trial. This outcome suggests that non-surgical interventions, coupled with personalized support, can effectively address initial barriers to CPAP adherence. Additionally, the persistence of side effects like nasal congestion and psychological difficulties underscores the need for comprehensive, multidisciplinary approaches to manage OSA treatment effectively (Ryan et al., 2009).

Our findings have several implications for research and clinical practice. The demonstrated effectiveness of patient-centric and technologically advanced approaches in improving CPAP adherence among initially resistant patients provides a valuable framework for developing tailored interventions. This study contributes to the ongoing discourse on optimal therapeutic strategies for OSA, emphasizing the importance of personalized medicine and continuous patient engagement.

However, our study has limitations, including the small sample size and the lack of marital status data (Goosmann et al., 2022), which could influence adherence. The short duration of the initial CPAP experience among participants also limits the generalizability of our findings. Future research should aim to include larger, more diverse populations and explore the long-term impacts of various patient-centric interventions on CPAP adherence.

In conclusion, our study supports the hypothesis that personalized approaches, continuous support, and technological advancements can significantly enhance CPAP adherence among patients initially resistant to therapy. These findings underscore the complexity of managing OSA and advocate for a nuanced, patient-centered approach to treatment, contributing to improved clinical outcomes and patient quality of life.

## Data availability statement

The original contributions presented in the study are included in the article/Supplementary material, further inquiries can be directed to the corresponding author.

## Ethics statement

The studies involving humans were approved by the Université Catholique de Louvain Number 2016/24NOV/503. The studies were conducted in accordance with the local legislation and institutional requirements. Written informed consent for participation was not required from the participants or the participants' legal guardians/next of kin in accordance with the national legislation and institutional requirements.

## Author contributions

GM: Writing – review & editing, Writing – original draft, Supervision, Software, Methodology, Investigation, Data curation, Conceptualization. JB: Investigation, Conceptualization, Writing – original draft, Writing – review & editing. DR: Writing – original draft, Writing – review & editing.
