# Supplementary material for: Revitalizing CPAP adherence: lessons from THN study in patients with hypoglossal nerve stimulators
Source: Front Sleep. 2024 Jul 5;3:1380373. doi: 10.3389/frsle.2024.1380373 (PMC12713931; doi:10.3389/frsle.2024.1380373)
Supplement: Supplementary file 1 [file Table_1.DOCX]

Supplemental material

**Table 3 :** Follow up of all the patients from the first visit of inclusion in the study of THN 1

and 2.

| Patients | Total number of visits during the study: | Hospitalization for PSG (Polysomnographies) during studie |
| --- | --- | --- |
| 1 | 7 | 15 |
| 2 | 3 | 12 |
| 3 | 20 | 15 |
| 4 | 19 | 12 |
| 5 | 14 | 14 |
| 6 | 20 | 13 |
| 7 | 17 | 15 |
| 8 | 26 | 11 |
| 9 | 8 | 13 |
| 10 | 9 | 13 |
| 11 | 7 | 12 |

The average number of medical visits requested by patients was 13 +/- 7 for a 3-year follow-up.

In this pilot study, frequent adjustments were performed during neurostimulation, and patients had to undergo numerous titration polysomnographies.
